# Supplementary material for: Experience modulates gaze behavior and the effectiveness of information pickup to overcome the inversion effect in biological motion perception
Source: PLoS One. 2025 Jan 28;20(1):e0317290. doi: 10.1371/journal.pone.0317290 (PMC11774361; doi:10.1371/journal.pone.0317290)
Supplement: Attachment — Submitted filename: PONE-D-24-39075 (1)KAS.pdf [file pone.0317290.s002.pdf]

PLOS ONE

Experience Modulates Visual Search Strategies and the Effectiveness of Information Pickup to Overcome the Inversion Effect in Biological Motion Perception

--Manuscript Draft--

**Manuscript Number:**	PONE-D-24-39075
**Article Type:**	Research Article
**Full Title:**	Experience Modulates Visual Search Strategies and the Effectiveness of Information Pickup to Overcome the Inversion Effect in Biological Motion Perception
**Short Title:**	Experience Modulates Visual Search and Information Pickup
**Corresponding Author:**	Xiaoye Michael Wang<br>University of Toronto<br>Toronto, CANADA
**Keywords:**	Biological motion Perception; Inversion effect; Vertical dance; Visuomotor experience; Kinematic specification of dynamics
**Abstract:**	The inversion effect in biological motion suggests that presenting a point-light display (PLD) in an inverted orientation impairs the observer's ability to perceive the movement. This impairment is thought to arise from the observer's unfamiliarity with the dynamic characteristics of inverted motion. A previous perceptual study showed that compared to typical and non-dancers, vertical dancers (VDs) were more sensitive to the artificial inversion of dance movements performed either in an upright or inverted orientation when provided with dynamic information. The heightened sensitivity was attributed to the unique visuomotor experience of VDs that modulates the visual search strategies, allowing them to overcome the inversion effect. The current study examined the eye movements of dancers with and without VD experience while they watched PLDs of both upright and inverted dance movements to detect if the displays were artificially inverted. The results replicated the previous study, showing that VDs were more sensitive in detecting inverted movements. Eye-tracking data further revealed that VDs had longer and more frequent fixations on the lower torso area, where the harness was placed when performing the air movement. Although traditional dancers also attended to the lower torso area, their lack of experience with performing the inverted movements while wearing the harness limited their ability to interpret the observed motion for detection of the inversion. These findings highlight the role of specialized visuomotor experience in enhancing biological motion perception and have implications for training techniques that leverage visual strategies to improve performance in complex or unfamiliar movement contexts.
**Order of Authors:**	Xiaoye Michael Wang
	Zhichen Feng
	Mingming Yang
	Jing Samantha Pan
	Margaret A Wilson
	Qin Zhu
**Additional Information:**	
**Question**	**Response**
**Financial Disclosure**<br><br>Enter a financial disclosure statement that describes the sources of funding for the work included in this submission. Review the submission guidelines for detailed requirements. View published research	The author(s) received no specific funding for this work.

Powered by Editorial Manager® and ProduXion Manager® from Aries Systems Corporation

articles from *PLOS ONE* for specific examples.<br><br>This statement is required for submission and **will appear in the published article** if the submission is accepted. Please make sure it is accurate.<br><br>**Funded studies**<br>Enter a statement with the following details:<br>• Initials of the authors who received each award<br>• Grant numbers awarded to each author<br>• The full name of each funder<br>• URL of each funder website<br>• Did the sponsors or funders play any role in the study design, data collection and analysis, decision to publish, or preparation of the manuscript?<br><br>Did you receive funding for this work?	
**Competing Interests**<br><br>Use the instructions below to enter a competing interest statement for this submission. On behalf of all authors, disclose any competing interests that could be perceived to bias this work—acknowledging all financial support and any other relevant financial or non-financial competing interests.<br><br>This statement is required for submission and **will appear in the published article** if the submission is accepted. Please make sure it is accurate and that any funding sources listed in your Funding Information later in the submission form are also declared in your Financial Disclosure statement.<br><br>View published research articles from *PLOS ONE* for specific examples.	The authors have declared that no competing interests exist.

Powered by Editorial Manager® and ProduXion Manager® from Aries Systems Corporation

**NO authors have competing interests**<br><br>Enter: *The authors have declared that no competing interests exist.*<br><br>**Authors with competing interests**<br><br>Enter competing interest details beginning with this statement:<br><br>*I have read the journal's policy and the authors of this manuscript have the following competing interests: [insert competing interests here]*<br><br>* typeset	
**Ethics Statement**<br><br>Enter an ethics statement for this submission. This statement is required if the study involved:<br><br>• Human participants<br>• Human specimens or tissue<br>• Vertebrate animals or cephalopods<br>• Vertebrate embryos or tissues<br>• Field research<br><br>Write "N/A" if the submission does not require an ethics statement.<br><br>General guidance is provided below. Consult the submission guidelines for detailed instructions. **Make sure that all information entered here is included in the Methods section of the manuscript.**	University of Wyoming Institutional Review Board (IRB) with written informed consent from the participants.

Powered by Editorial Manager® and ProduXion Manager® from Aries Systems Corporation

**Format for specific study types**<br><br>**Human Subject Research (involving human participants and/or tissue)**<br>• Give the name of the institutional review board or ethics committee that approved the study<br>• Include the approval number and/or a statement indicating approval of this research<br>• Indicate the form of consent obtained (written/oral) or the reason that consent was not obtained (e.g. the data were analyzed anonymously)<br><br>**Animal Research (involving vertebrate animals, embryos or tissues)**<br>• Provide the name of the Institutional Animal Care and Use Committee (IACUC) or other relevant ethics board that reviewed the study protocol, and indicate whether they approved this research or granted a formal waiver of ethical approval<br>• Include an approval number if one was obtained<br>• If the study involved *non-human primates*, add *additional details* about animal welfare and steps taken to ameliorate suffering<br>• If anesthesia, euthanasia, or any kind of animal sacrifice is part of the study, include briefly which substances and/or methods were applied<br><br>**Field Research**<br><br>Include the following details if this study involves the collection of plant, animal, or other materials from a natural setting:<br>• Field permit number<br>• Name of the institution or relevant body that granted permission	
**Data Availability**<br><br>Authors are required to make all data underlying the findings described fully available, without restriction, and from the time of publication. PLOS allows rare exceptions to address legal and ethical concerns. See the PLOS Data Policy and FAQ for detailed information.	No - some restrictions will apply

A Data Availability Statement describing where the data can be found is required at submission. Your answers to this question constitute the Data Availability Statement and **will be published in the article**, if accepted.<br><br>**Important:** Stating 'data available on request from the author' is not sufficient. If your data are only available upon request, select 'No' for the first question and explain your exceptional situation in the text box.<br><br>Do the authors confirm that all data underlying the findings described in their manuscript are fully available without restriction?	
**Describe where the data may be found in full sentences. If you are copying our sample text, replace any instances of XXX with the appropriate details.**<br><br>• If the data are **held or will be held in a public repository**, include URLs, accession numbers or DOIs. If this information will only be available after acceptance, indicate this by ticking the box below. For example: *All XXX files are available from the XXX database (accession number(s) XXX, XXX.).*<br>• If the data are all contained **within the manuscript and/or Supporting Information files**, enter the following: *All relevant data are within the manuscript and its Supporting Information files.*<br>• If neither of these applies but you are able to provide **details of access elsewhere**, with or without limitations, please do so. For example:<br><br>*Data cannot be shared publicly because of [XXX]. Data are available from the XXX Institutional Data Access / Ethics Committee (contact via XXX) for researchers who meet the criteria for access to confidential data.*<br><br>*The data underlying the results presented in the study are available from (include the name of the third party*	Data cannot be shared publicly because of a lack of participants' consent on sharing their data.<br><br>Data could be made available upon request to Nichole Person (Research Compliance Coordinator, Office of Research Economic Development, University of Wyoming) at njperson@uwyo.edu.

Powered by Editorial Manager® and ProduXion Manager® from Aries Systems Corporation

and contact information or URL).<br>• This text is appropriate if the data are owned by a third party and authors do not have permission to share the data.<br><br>* typeset	
Additional data availability information:	

Powered by Editorial Manager® and ProduXion Manager® from Aries Systems Corporation

Click here to access/download;Manuscript;Experience Modulates Visual Search V5.docx

Experience Modulates Visual Search Strategies and the Effectiveness of Information Pickup to Overcome the Inversion Effect in Biological Motion Perception

Xiaoye Michael Wang [a]

Zhichen Feng [b]

Mingming Yang [b]

Jing Samantha Pan [c, d]

Margaret A. Wilson [e]

Qin Zhu [b]

RUNNING HEAD: Experience Modulates Visual Search and Information Pickup

a. Faculty of Kinesiology and Physical Education, University of Toronto, Toronto, ON, CANADA
b. Division of Kinesiology and Health, University of Wyoming, Laramie, WY, USA
c. Department of Psychology, Sun Yat-sen University, Guangzhou, CHINA
d. Guangdong Provincial Key Laboratory of Social Cognitive Neuroscience and Mental Health, Guangzhou, CHINA
e. Department of Theatre and Dance, University of Wyoming, Laramie, WY, USA

Corresponding Author: Xiaoye Michael Wang. Email: michaelwxy.wang@utoronto.ca

Abstract

The inversion effect in biological motion suggests that presenting a point-light display (PLD) in an inverted orientation impairs the observer's ability to perceive the movement. This impairment is thought to arise from the observer's unfamiliarity with the dynamic characteristics of inverted motion. A previous perceptual study showed that compared to typical and non-dancers, vertical dancers (VDs) were more sensitive to the artificial inversion of dance movements performed either in an upright or inverted orientation when provided with dynamic information. The heightened sensitivity was attributed to the unique visuomotor experience of VDs that modulates the visual search strategies, allowing them to overcome the inversion effect. The current study examined the eye movements of dancers with and without VD experience while they watched PLDs of both upright and inverted dance movements to detect if the displays were artificially inverted. The results replicated the previous study, showing that VDs were more sensitive in detecting inverted movements. Eye-tracking data further revealed that VDs had longer and more frequent fixations on the lower torso area, where the harness was placed when performing the air movement. Although traditional dancers also attended to the lower torso area, their lack of experience with performing the inverted movements while wearing the harness limited their ability to interpret the observed motion for detection of the inversion. These findings highlight the role of specialized visuomotor experience in enhancing biological motion perception and have implications for training techniques that leverage visual strategies to improve performance in complex or unfamiliar movement contexts.

Keywords: Biological motion Perception, Inversion effect, Vertical dance, Visuomotor experience, Kinematic specification of dynamics.

Introduction

As terrestrial animals, gravity has been an indispensable element in human's evolutionary past, present, and future (Morey-Holton, 2003). Its significance not only lies in a fallen apple mentioned in a textbook, but it also manifests in learning and controlling human movements. Any human movement is the result of the entire force environment, the presence of gravity is a key aspect in determining how a person moves. The influence of gravity (or a lack thereof) can be readily seen in how different astronauts move in space, where performing motor tasks in a microgravity environment without feet on the ground presents a new challenge. Past research has shown that human observers could readily take advantage of the dynamic (force) information embedded in human movements to perform various types of perceptual judgment, such as the actor's gender (Barclay et al., 1978; Troje et al., 2006) and identity (Cutting & Kozlowski, 1977; Troje et al., 2005; Wang, 2023), as well as the effect of actions (Zhu & Bingham, 2014). To control for other visual features (e.g., configuration information such as shape and size) that convey information about the actor and the action, point-light displays (PLD) are used to isolate the dynamic and configural information of the movement (Johansson, 1973, 1976). PLDs represent a person or an animated object using a series of moving points that are placed at different major joints (e.g., shoulders, elbows, wrists, pelvis, knees, and ankles). According to the *kinematic specification of dynamics* theory (Runeson & Frykholm, 1983), how an object moves (kinematics) is determined by the object's entire force environment (dynamics), including gravity. Therefore, altering the force environment would in turn perturb how an object moves, as in the case of an astronaut in space.

Reducing gravitational force is not the only method to perturb the force environment in which a person moves. Studies have shown that simply inverting the PLD could impair the

observer's ability to perceive biological motion, termed the *inversion effect* of biological motion (Pavlova & Sokolov, 2000; Shipley, 2003; Sumi, 1984). For instance, Shipley (2003) used PLD[illegible]o present a person walking where the display was either in the movement's canonical orientation or upside down. In this context, the artificial inversion also inverted the direction of gravity in the display, altering the embedded dynamics of the movement. Results showed that the artificial inversion reduced the participants' ability to identify meaningful movement patterns in the display, suggesting the importance of dynamic information in specifying natural movements.

While the inversion effect yields impairment to biological motion perception, a recent study suggested that visuomotor experience with inverted movements could help the observers to overcome this adverse effect (Wang et al., 2022). This study focused on the unique visuomotor experience of vertical dancers (VD), as compared to typical (TD) and non-dancers (ND). Vertical dance is a form of aerial modern dance in which the dancers are suspended in mid-air wearing a harness around the waist and hips and perform various movements either in an upright or upside-down orientation. As a result, VDs have both extended visual experience of observing others performing movements in an inverted orientation and the motor and proprioceptive experience of performing such movements themselves. In this study, a series of dance movements were recorded either on the ground or in the air before being artificially inverted in PLDs. Then, a mixture of original and inverted PLDs was presented either in their entirety (i.e., with sufficient dynamic information) or as a one-second clip that only depicts the final form of the movement (i.e., with limited dynamic information) for observers to detect the artificial inversion. Results showed that, while TD and ND had difficulties discerning the artificial inversion of the movements performed in the air, VD could readily do so when given sufficient dynamic information.

Unlike walking, dance movements are more complex, involving the coordination between different body segments and using both upper and lower limbs to demonstrate diverse postures. With their feet off the ground and body suspended in the air, vertical dancers have more degrees of freedom to use their trunks and limbs for creative performance. Therefore, the unexpectedly greater range of motion associated with the trunk and limbs in vertical dance, particularly in the inverted orientation, could have confused observers who typically see movements performed on the ground, limiting their ability to judge and interpret the vertical dance movements. Although Wang and colleagues (2022) demonstrated that VDs were able to use dynamic information in detecting the inversion effect, the source of such dynamic information in the display based on which the judgment was made remains unclear. Could the information come from the upper limb, trunk, and lower limb, or it is a relationship among them? Identifying the source of dynamic information in vertical dance could not only advance the scientific understanding of human movement perception but also yield insights into the ways through which dancers can improve their performance (e.g., enhance the expressiveness and impact of their performances by focusing on body segments that convey movement dynamics).

Visual search behaviors are dictated by five factors, including bottom-up salience, top-down feature guidance, scene structure and meaning, the previous history of search over timescales, and the relative value of the targets and distractors (Wolfe & Horowitz, 2017). For instance, radiologists rely on years of education and training to adopt a global-focal search strategy, allowing them to quickly detect salient abnormalities in medical images by matching these features to structures stored in their memory (Ganesan et al., 2018). Similarly, due to their extensive experience performing and observing dance movements in both upright and inverted orientations, VDs are expected to be more attuned to the dynamic information in the display, which helps them

overcome the inversion effect. The question then becomes: What specific visual information do VDs utilize during the task that enables them to make more accurate judgments?

Eye-tracking technology is commonly employed to study visual search behaviors, allowing researchers to differentiate between the search patterns of experts and novices and identify the visual information picked up during the process. Stevens et al. (2010) examined the fixations and saccades of expert and novice dance observers. They found that the fixation duration was shorter, and saccades were faster, for experts than novices, indicative of a more deliberate and efficient search strategy. Moreover, what the observers fixated on also differed: experts attended mostly to the head of the dancers, while novices attended equally to the head, neck, torso, and arms. These results were replicated by Ponmanadiyil and Woolhouse (2018), who compared the visual search behaviors between experienced and inexperienced dancers when observing Indian Bharatanatyam dance. Given that VDs are more capable than to non-VDs are better than non-VDs at detecting inverted dance movements, it raises the question of whether dancers with and without VD experience employ distinct visual search strategies when tasked with detecting artificially inverted movements.

The current study aimed to extend the findings from Wang et al. (2022) by incorporating eye-tracking measures to identify the sources of dynamic information that VDs rely on when detecting inverted movements. It was hypothesized that, compared to typical and non-dancers, VDs would engage in shorter fixations and faster saccades, reflective of a more efficient search strategy, due to their extended visuomotor experience with inverted dance movements. Moreover, the analysis of fixation areas was expected to reveal the specific sources of dynamic information that enable VDs to make accurate perceptual judgments regarding the inversion of movements.

Methods

Participants

37 adults participated in this experiment, including 12 non-dancers (ND; age M = 23.00 years, SD = 2.22), 13 traditional dancers (TD; age M = 22.17 years, SD = 1.47), and 12 vertical dancers (VD; age M = 32.90 years, SD = 18.56). TDs had an average of 12.25 years of dance experience, whereas VDs had an average of 9.82 years of vertical dance experience. The experiment was in accordance with the Declaration of Helsinki and was approved by the University of Wyoming Institutional Review Board (IRB). All participants provided informed consent before participation.

Stimuli and Apparatus

The visual stimuli used in this study were identical to that of Wang et al. (2022). A male and a female professional vertical dancer performed a series of dance movements while being fitted with 13 optical markers. The markers' 3D positions were captured using a Vicon Motion Capture system with eight Bonita Optical cameras sampling at 160 Hz. The markers correspond to 13 major joints, including the head, the left and right shoulders, elbows, wrists, hips, knees, and ankles. The 3D coordinates of each movement were projected onto a 2D plane to generate the animation. Both dancers provided their informed consent for the motion capture and the subsequent use of the motion capture data for the experiment.

Each dancer performed 20 movements, where 10 movements were performed on the ground and the other 10 were performed in the air while being suspended in a harness (Table 1). Figure 1 demonstrates the basic distinctions between different types of movements. The air and ground movements were selected such that each movement pair is similar, if not identical, in form

(i.e., same configural information), but only differ in terms of their recording conditions (i.e., on the ground or in the air). Moreover, of the ten ground-air pairs, five pairs' canonical orientations were identical (congruent, i.e., both the ground- and air-based movements were performed in an upright/inverted orientation), whereas the other five pairs had opposite canonical orientations (incongruent, i.e., the ground movement was performed in an upright/inverted orientation while the air movement was performed in an inverted/upright orientation). Movements performed by one dancer were kept in their original sequence (mean duration = 4.05 s, SD = 1.10, range = [1.25, 5.94] s). Presented in their entirety, these movements provided complete dynamic information. In contrast, movements performed by the other dancer were trimmed such that only the final form of the movement was available for one second, where the dancer remained relatively static. These movements only provided limited dynamic information and required the participants to rely more on the configuration information to perform the judgment.

Ground	Air	Congruency
Arabesque Fondu	Inverted Arabesque Fondu	Incongruent
Attitude devant over forced arch/arms forward	Inverted attitude devant/arms forward	Incongruent
Développé à la seconde	Inverted développé à la seconde	Incongruent
Italian Pas de Chat	Inverted Pas de Chat	Incongruent
X – Plié/extend	Inverted X – Plié/extend	Incongruent
Attitude spiral	Side-lying attitude	Congruent
Cambre Back B+	High release one leg attitude	Congruent
Plank to V-sit	Plank to V-sit	Congruent
Supported handstand flex/point	Inversion hook foot	Congruent
Side Plank – hip lower and lift	Side-lying limbs lower/lift	Congruent

Table 1. List of dance movements

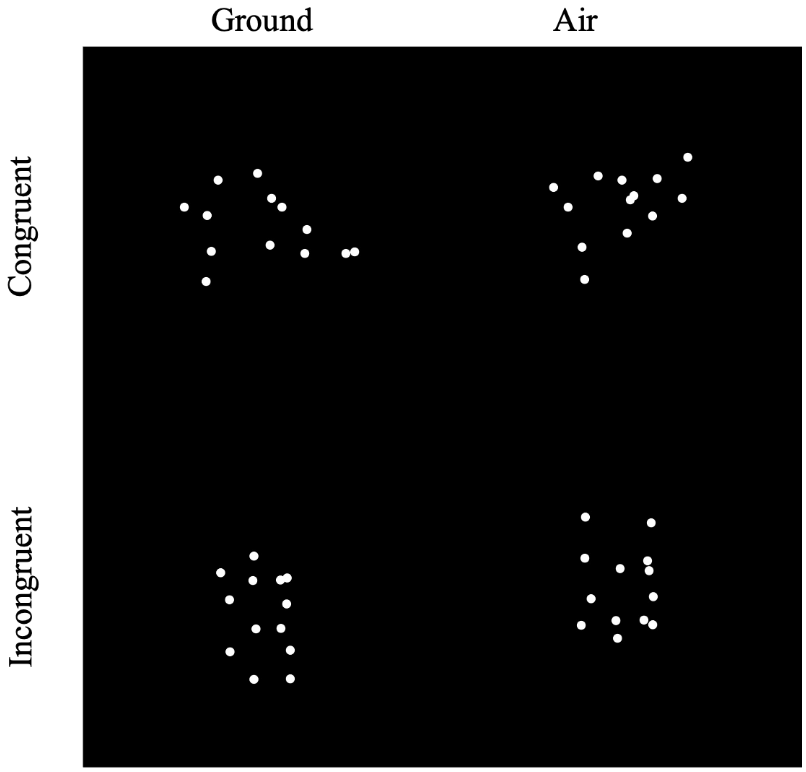

Figure 1. Illustration of the ground and air movements when the movement pair was incongruent (top; ground: side Plank – hip lower and lift; air: side lying limbs lower/lift) or congruent (bottom; ground: X – Plié/extend; air: Inverted X – Plié/extend). More details can be found in a video demo (https://youtu.be/6vLQgQpy-uc) from the previous study (Wang et al., 2022).

The experiment was presented on a Dell 22-inch monitor (E2222H) with a 1680 × 1050 resolution. The monitor was placed 57 cm in front of the participant while the participant rested their head on a chinrest. The experiment was coded in Python using functionalities from PsychoPy (Peirce et al., 2019). Eye tracking was performed using Tobii Pro Fusion. The eye tracker was attached to the bottom of the monitor. Raw eye tracking data were collected using PyGaze, a Python-based open-source toolbox that offers an interface with Tobii devices (Dalmaijer et al., 2014).

Procedure

After providing their informed consent, the participants were first introduced to the task using a laptop computer. They were presented with a pair of ground-air movements that were not

used in the actual experiment. The movement animations represented each joint as a 10-pixel white circle displayed against a black background (Figure 1). The tutorial used these movements to illustrate the difference between ground and air movements and between the different canonical orientations (upright or inverted). The participants were told that the displayed movements could be artificially inverted, and their task was to identify whether the artificial inversion had occurred in an animation. They responded by pressing the F key for inverted and the J key for not inverted. Then, the participants did four practice trials using the demonstration movements and received feedback.

After the tutorial, the participants were seated in front of the monitor and placed their chin on a custom-made chinrest. The experiment started with calibrating the eye tracker, which was performed using PyGaze's calibration method. The subsequent perceptual judgment task was blocked by Dynamic Type (complete or limited), presented in a random order. In each block, there were 20 movements (5 movements × 2 Recording Types [ground or air] × 2 Congruency [congruent or incongruent]) that were either presented as-is or artificially inverted, resulting in a total of 40 trials, presented in random order. In each trial, each movement animation was repeated three times, after which the participants were asked to determine whether the animation was artificially inverted. Combining two blocks, the entire experiment contained 80 trials and took approximately 30 minutes to complete.

Data Analysis

Perceptual Judgment

Perceptual judgment was analyzed using signal detection theory (Macmillan & Creelman, 2005) to derive the participants' sensitivity to artificial inversion. For each participant, trials were

grouped based on Dynamic Types (complete or limited), Congruency (congruent or incongruent), and Recording Types (ground or air). Each group contained 10 trials with responses of yes for the displayed movement was artificially inverted and no for the movement was not inverted. The artificial inversion was considered as the signal and therefore, a hit represents a yes response when the movement was artificially inverted, whereas a false alarm corresponds to a yes response when the movement was not inverted. The sensitivity, d', was calculated as the difference in z-scores between hit and false alarm (Stanislaw & Todorov, 1999). A d' value greater than 0 indicates the participants were able to identify the artificial inversion above the chance level, whereas a negative d' value suggests that the participants considered the movements displayed in their canonical orientation as being artificially inverted. Subsequently, a mixed-design analysis of variance was used to analyze the effects of one between-subject factor of Experience (three levels: ND, TD, and VD) and three within-subject factors of Dynamic Type (two levels: complete and limited), Congruency (two levels: congruent and incongruent), and Recording Type (two levels: ground and air) on the derived d' values. Greenhouse-Geisser corrections were applied to effects that violate the sphericity assumption and were indicated by the decimal degrees of freedom values.

Eye Tracking

Raw eye-tracking data were processed using PyTrack, a Python-based eye-tracking analysis toolkit (Ghose et al., 2020). Fixation threshold was set at 2° with a minimum duration of 100 ms and saccades were identified based on a velocity threshold of 60°/s with a minimum duration of 30 ms. Fixation frequency and duration as well as saccade frequency were derived for each trial. To further examine the relationship between fixation points and the PLD, an additional spatial analysis of the gaze patterns was performed. The locations of the fixation and the locations

of each point in PLD were normalized based on the screen's resolution, resulting in values between 0 and 1. The dancer's center of mass was approximated as the centroid between the two shoulders and the two hip joints, referred to as the torso center. To derive a spatial measure for the relationship between fixation and the PLD, the Euclidean distance between each joint and the fixation point was calculated for each animation frame during the fixation period. Then, the mean distances, referred to as the mean normalized distance, across all fixation periods were calculated for each joint in every trial.

Based on findings of a previous study (Wang et al., 2022), it was expected that, due to their extended visuomotor experience, VDs were more sensitive to the inversion of air movements than NDs and TDs when given complete dynamic information and regardless of the movement's congruency. In this context, the goal of eye tracking analysis is to investigate the source of the visual information in the animation that VDs focused on to achieve higher sensitivity. Therefore, statistical analysis of eye tracking data, including gaze events and the mean normalized distance, will only use data from the complete dynamic condition and will not include Congruency as a separate factor. Therefore, for the gaze event measures (i.e., saccade frequency, fixation frequency, and fixation duration), a series of mixed-design ANOVA with one between-subject factor (Experience) and one within-subject factor (Recording Type) was used. For the normalized fixation distance, an additional within-subject factor, Joints, was introduced to the mixed ANOVA.

Results and Discussion

Perceptual Judgment

Table 2 shows the mixed-design ANOVA results for d'. Because the current study involved direct replication of a previous study (Wang et al., 2022), discussions of perceptual judgment results will primarily focus on significant effects related to Experience. For the significant main effect of Experience, VD (mean = 0.84, SE = 0.095) had significantly greater d' than ND (mean = 0.36, SE = 0.095, $p < 0.01$), while VD's difference with TD (mean = 0.56, SE = 0.092) failed to reach the traditional level of significance ($p = 0.097$). There was no difference between TD and ND ($p = 0.30$). The current finding is consistent with the general patterns of results reported earlier, where due to their extended visuomotor experience with inverted movements, VDs demonstrated an advantage over TDs and NDs with regard to differentiating between physically and artificially inverted movements.

Effects	DF1	DF2	F	η_p^2	p	
Experience	2	34	6.47	0.28	< 0.01	**
Recording type	1	34	59.17	0.64	< 0.001	***
Experience × Recording type	2	34	8.29	0.33	< 0.01	**
Congruency	1	34	0.09	0.00	0.77	
Experience × Congruency	2	34	1.06	0.06	0.36	
Dynamic type	1	34	40.12	0.54	< 0.001	***
Experience × Dynamic type	2	34	6.69	0.28	< 0.01	**
Recording type × Congruency	1	34	18.39	0.35	< 0.001	***
Experience × Recording type × Congruency	2	34	5.33	0.24	< 0.01	**
Recording type × Dynamic type	1	34	1.38	0.04	0.25	
Experience × Recording type × Dynamic type	2	34	0.82	0.05	0.45	
Congruency × Dynamic type	1	34	10.35	0.23	< 0.01	**
Experience × Congruency × Dynamic type	2	34	0.53	0.03	0.59	

Recording type × Congruency × Dynamic type	1	34	4.81	0.12	< 0.05	*
Experience × Recording type × Congruency × Dynamic type	2	34	1.01	0.06	0.37	

Table 2. Results of the mixed-design ANOVA on d'. * $p < 0.05$, ** $p < 0.01$, *** $p < 0.001$.

For the significant main effect of Recording Type, sensitivity to the artificial inversion of ground movement (mean = 1.27, SE = 0.10) was significantly greater than that of air movement (mean = -0.10, SE = 0.11, $p < 0.001$). As expected, participants across all experience levels were more familiar with movements performed on the ground and, therefore, were more sensitive to the artificial inversion of ground movements. Interestingly, the significant two-way interaction between Experience and Recording Type and the three-way interaction between Experience, Recording Type, and Congruency revealed the impact of visuomotor experience on perceptual judgment. Because the significant three-way interaction encompasses the two-way interaction, only the post-hoc analysis of the three-way interaction will be discussed (Figure 2). Experience was used as a simple effect to reduce the total number of post-hoc pairwise comparisons. For the congruent movements, where the air and ground movement counterparts were performed in the same canonical direction, there was no difference between any Experience pairs when the movement was performed on the ground (VD – TD: $p = 0.98$; VD – ND: $p = 0.91$; TD – ND: $p = 0.97$). When the movement was performed in the air, VD (mean = 0.63, SE = 0.16) showed greater sensitivity than TD (mean = 0.04, SE = 0.16, $p < 0.05$) and ND (mean = 0.08, SE = 0.16, $p = 0.06$), albeit the latter only approached the traditional level of significance. For the incongruent movements, VD (mean = 0.71, SE = 0.36) had significantly greater sensitivity than TD (mean = -0.73, SE = 0.35, $p < 0.05$) and ND (mean = -1.34, SE = 0.36, $p < 0.01$) when the movement was performed in the air. The noticeable negative d' values for TD and ND are indicative that both

groups mistook un-inverted air movements (in an upside-down canonical orientation) as being artificially inverted. This reflected the general unfamiliarity of TD and ND with vertical dance and their general bias towards movement performed in an upright orientation. Because the canonical orientation of the incongruent air movements was upside down, the large negative d' values suggest that TD and ND simply considered the upright orientation as the movement's canonical orientation and judged every display with an upside-down dancer as being artificially inverted, regardless of the actual orientation in which movement was performed. For the incongruent ground movements, VD (mean = 1.01, SE = 0.22) had significantly lower sensitivity than TD (mean = 1.96, SE = 0.21, $p < 0.05$) and ND (mean = 1.79, SE = 0.22, $p < 0.05$). This could also reflect the stronger bias of TD and ND where they generally considered inverted movements as being artificially inverted from the ground movements and the lack of a similar bias of VD.

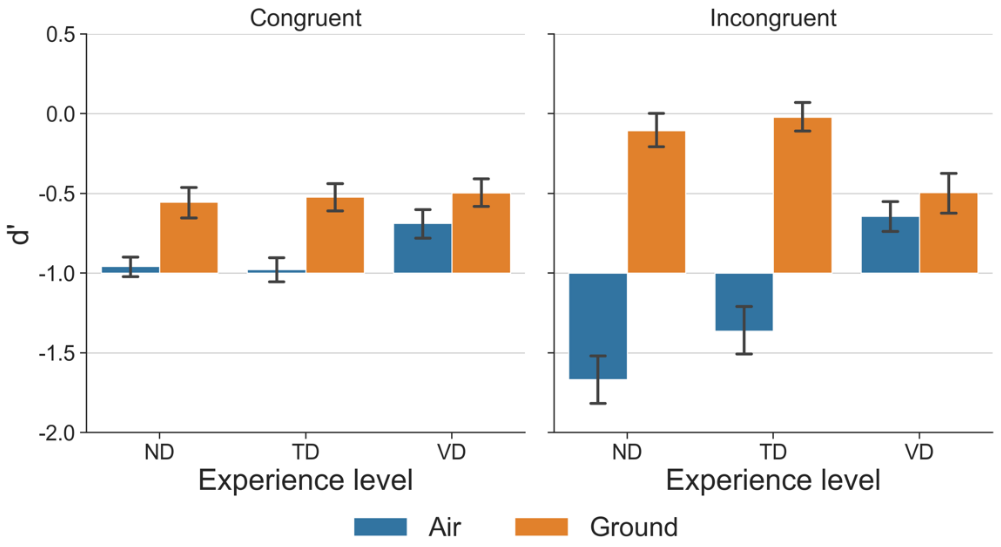

Figure 2. Mean d' as a function of Experience (*x*-axis: ND: non-dancers, TD: typical dancers, VD: vertical dancers) for different Recording Types (hue) for the congruent (left) and incongruent (right) air-ground movement pairs. Error bars represent the standard error of the means.

For the main effect of Dynamic Type, sensitivity in the Complete condition (mean = 0.87, SE = 0.08) was significantly higher than that in the Limited condition (mean = 0.30, SE = 0.05, p < 0.001). Importantly, the significant interaction between Experience and Dynamic Type showed how well different participant groups utilized the dynamic information. For the Limited condition, no difference was observed between any Experience pair (VD – TD: p = 0.71; VD – ND: p = 0.82; TD – ND: p = 0.98). However, for the Complete condition, VD (mean = 1.32, SE = 0.15) had greater sensitivity than TD (mean = 0.85, SE = 0.14, p = 0.073) and ND (mean = 0.43, SE = 0.15, p < 0.001), albeit the former only approached the traditional level of significance. This finding suggests that, compared to TDs and NDs who did not have any experience with vertical dance, VDs were more capable of utilizing the dynamic information in the display to perform correct judgment, which replicated previous results (Wang et al., 2022).

Eye Tracking Analysis

The primary goal of this study was to examine gaze patterns to figure out how VDs utilized dynamic information to distinguish artificial inversions in air and ground movements. To this end, only data from the Complete condition were used. A series of mixed-design ANOVAs with Experience (between-subject) and Recording Type (within-subject) as factors were performed for the number of saccades, number of fixations, and fixation duration. Congruency was not included in the analysis because it is more relevant to the intrinsic bias of each participant group, instead of their ability to use the dynamic information in the display.

For the number of saccades, there was only a significant main effect of Recording Type, $F(1,34) = 20.89, p < 0.001, \eta_p^2 = 0.38$, while neither Experience, $F(2,34) = 1.22, p = 0.31, \eta_p^2 = 0.07$, nor the interaction, $F(2,34) = 0.02, p = 0.98, \eta_p^2 = 0.001$, were significant.

There were significantly more saccades with the air movement (mean = 16.9, SE = 0.95) than with the ground movement (mean = 15.2, SE = 0.78, $p < 0.001$). This finding suggests an increased level of visual exploration in the air condition, which also corresponded to an increased task difficulty. In other words, because of the air movements' uniqueness, the participants across all experience levels engaged in more frequent visual searches to identify relevant visual information to perform the judgment. For the number of fixations, there was again only a significant main effect of Recording Type, $F(1, 34) = 34.62, p < 0.001, \eta_p^2 = 0.50$, but not Experience, $F(2, 34) = 0.01, p = 0.99, \eta_p^2 < 0.001$, or the interaction, $F(2, 34) = 0.34, p = 0.72, \eta_p^2 = 0.02$. There were more fixations with the air movements (mean = 15.4, SE = 0.68) than with the ground movement (mean = 13.9, SE = 0.58, $p < 0.001$). This finding is consistent with the increased saccade frequency with the air movement, further demonstrating more visual exploration.

Finally, for the fixation duration, Recording Type was significant, $F(1, 34) = 5.00, p < 0.05, \eta_p^2 = 0.13$. Although the main effect of Experience was not significant, $F(2, 34) = 0.74, p = 0.48, \eta_p^2 = 0.04$, the interaction between Experience and Recording Type was, $F(2, 34) = 3.68, p < 0.05, \eta_p^2 = 0.18$. As with other gaze metrics, fixation duration for air movements (mean = 401 ms, SE = 9.60) was significantly greater than that for ground movements (mean = 394 ms, SE = 9.27, $p < 0.05$). More crucially, for the significant interaction, there was no difference between the air and ground movements for TDs ($p = 0.51$) or NDs ($p = 0.19$). However, for VDs, the fixation duration for the air movements (mean = 403 ms, SE = 16.84) was significantly longer than that for the ground movements (mean = 384 ms, SE = 16.26, $p < 0.01$). The longer fixation duration for VDs with the air movement indicates that VDs concentrated on certain features of the air displays more than in the ground displays. In conjunction with their general high sensitivity to the artificial inversion in the air movement and a lack of difference in their saccade

and fixation frequencies, this finding implies that VDs were able to identify the potential area of interest more readily in the display and extrapolate the relevant information that revealed the canonical orientation of the movement. Subsequently, the spatial patterns of the fixation were analyzed to explore the sources of visual information that VDs relied on to perform the judgment.

Spatial Analysis

The spatial analysis of gaze patterns focused on delineating the relationship between fixation points and the joints in PLD. The mean normalized distances between the fixation point and each joint (including the extrapolated torso center) were derived. Similar to the gaze event analysis, only data from the Complete dynamic condition were used. A mixed-design ANOVA was performed with one between-subject factor (Experience) and two within-subject factors (Recording Type and Joint). There was a significant main effect of Experience, $F(2, 34) = 5.98, p < 0.01, \eta_p^2 = 0.26$. Post-hoc pairwise comparisons showed that the normalized distance for NDs (mean = 0.18, SE = 0.0059) was significantly greater than that for VDs (mean = 0.16, SE = 0.0059, $p < 0.01$) and that for TDs (mean = 0.16, SE = 0.0056, $p < 0.05$). There was no difference between VDs and TDs ($p = 0.65$). This finding indicates that, on average, VDs and TDs tended to focus more on the movement of specific joints when performing the perceptual judgment. There was also a significant main effect of Recording Type, $F(1, 34) = 11.27, p < 0.01, \eta_p^2 = 0.25$. The average fixation locations were closer to each animated joint in the Air condition (mean = 0.16, SE = 0.0029) than that in the Ground condition (mean = 0.18, SE = 0.0046, $p < 0.01$). This result suggests that with the more difficult air movements, the participants tended to focus more on the dancer's joints.

The main effect of Joint was also significant, $F(1.50, 51.07) = 928.33, p < 0.001, \eta_p^2 = 0.97$. Noticeably, the torso center had the smallest distance (mean = 0.11, CI = [0.10, 0.13]), followed by the right and left hips (right: mean = 0.12, CI = [0.11, 0.14]; left: mean = 0.12, CI = [0.11, 0.14]). This result is congruent with the characteristics of the air and ground movements. To perform the movements in the air, the dancers had to use a harness that necessarily fixed their lower torso area. Moreover, during movement recording, the dancers were instructed to minimize oscillatory movement. As a result, compared to their ground counterparts, the dancers' lower torso would remain relatively stable, which was reflected through the movement of the derived torso center and the hips.

Finally, although the interaction between Experience and Joint was not significant, $F(3.00, 51.07) = 2.71, p = 0.055, \eta_p^2 = 0.14$, the interaction between Recording Type and Joint, $F(2.11, 71.69) = 84.96, p < 0.001, \eta_p^2 = 0.71$, and the three-way interaction between Experience, Recording Type, and Joint, $F(4.22, 71.69) = 3.39, p < 0.05, \eta_p^2 = 0.17$, were both significant. Given the significant three-way interaction, post-hoc analysis was focused on examining the difference between experience levels (i.e., using Experience as a simple effect) within each Recording Type and at each Joint (Table 3). For the 14 joints included in the analysis, we divided them into upper and lower body.

		Ground			Air		
	Joints	ND - TD	ND - VD	TD - VD	ND - TD	ND - VD	TD - VD
Upper	Head	< **0.05**	< **0.05**	0.56	< **0.01**	< **0.01**	0.97
	Torso Center	0.093	< **0.05**	0.71	0.11	< **0.05**	0.88
	Shoulder (L)	< **0.05**	< **0.01**	0.53	< **0.01**	< **0.05**	1.00
	Shoulder (R)	0.085	< **0.01**	0.56	< **0.05**	< **0.05**	1.00
	Elbow (L)	< **0.05**	< **0.01**	0.68	< **0.05**	< **0.05**	1.00
	Elbow (R)	0.11	< **0.05**	0.57	< **0.05**	< **0.05**	0.93
	Wrist (L)	< **0.05**	< **0.01**	0.67	< **0.05**	< **0.05**	1.00
	Wrist (R)	0.088	< **0.01**	0.5	< **0.05**	< **0.05**	0.96

Lower	Hip (L)	0.07	**< 0.05**	0.89	0.47	0.15	0.72
	Hip (R)	0.16	0.050	0.82	0.53	0.14	0.63
	Knee (L)	0.094	0.057	0.96	0.89	0.17	0.34
	Knee (R)	0.23	**< 0.05**	0.68	0.91	0.14	0.27
	Ankle (L)	0.16	0.12	0.98	1.00	0.17	0.16
	Ankle (R)	0.31	0.067	0.66	1.00	0.19	0.16

Table 3. P-values for the post-hoc comparisons of the three-way interaction between Experience, Recording Type, and Joints with Experience as the simple effect.

As shown in **Error! Reference source not found.**, the significantly longer fixation distance for NDs observed previously was generally reflected on a joint level for the air and ground movements. For ground movements, VDs fixated at locations closer to all the joints associated with the upper body compared to NDs ($p < 0.05$ or $p < 0.01$), whereas the difference between TDs and NDs was less consistent across different upper body joints where differences of some joints were not significant (i.e., torso center, shoulder (R), elbow (R), and wrist (R). Importantly, VDs, but not TDs, are fixated closer to the torso center and the left and right hips than NDs. For air movements, the difference between TDs and NDs for the upper body joints became more consistent, similar to the difference between VDs and NDs. However, the difference between TD and ND for the derived torso center failed to reach significance, whereas that between VDs and NDs did. Moreover, there was no difference between any experience pair in terms of the fixation distance to the left and right hips. Combined with VDs' greater sensitivity to the inverted air movements compared to TDs and NDs, this finding indicates that VDs focused more on the overall dynamic patterns of the torso movement, extrapolated based on the key torso joints (shoulders and hips) when viewing the PLD. Furthermore, although there was no significant difference between TDs and VDs in terms of their fixation locations, due to a lack of experience with vertical dance, TDs had an intrinsic bias towards ground movements. As a result, even though TDs may potentially be able to identify the relevant visual information from the display based on their

comparable visual search patterns as VDs, they still failed to correctly identify the artificial inversion, as demonstrated in the d' analysis.

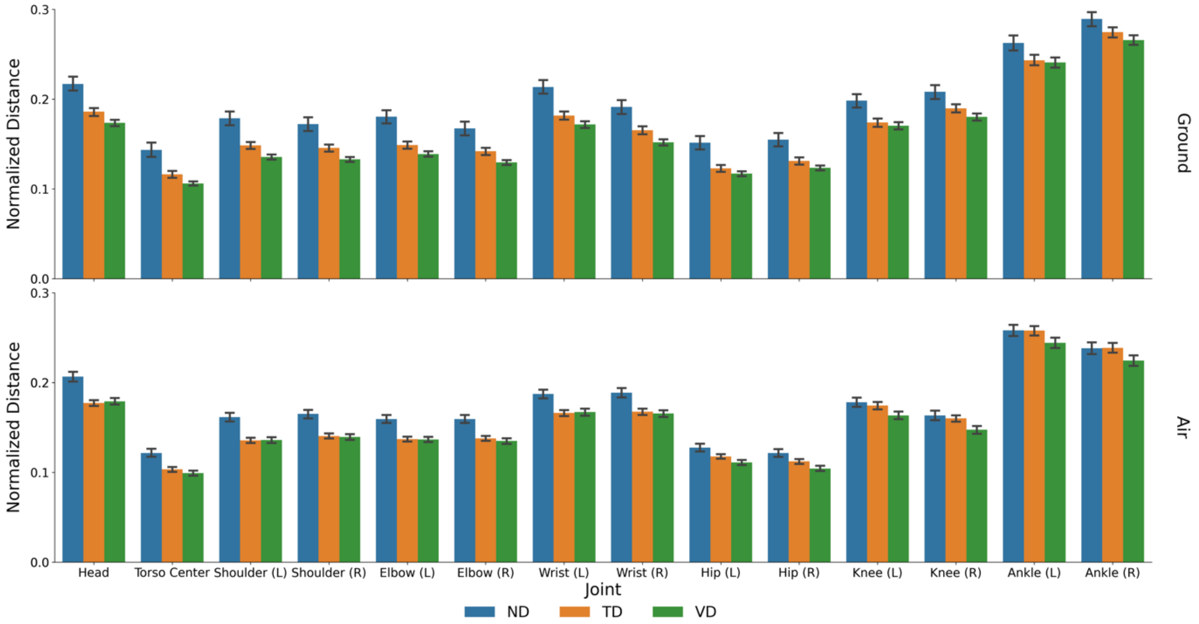

Figure 3. Mean normalized distance between the fixation point and each joint (*x*-axis) represented by the point-light display for participants with different experience levels (hue; ND: non-dancers, TD: typical dancers, VD: vertical dancers) for the ground (top) and air (bottom) movements. The torso center was derived as the midpoint of the two shoulders and the two hip joints. Error bars represent the standard error of the means.

General Discussion

Pondering the inversion effect in biological motion perception, the current study examined whether the extended visuomotor experience with inverted movements could help the observer restore the human movement perception that was contaminated by the inversion. Working with professional VDs, Wang et al. (2022) systematically manipulated the orientation of dance movements in PLDs and showed that only VDs, but not TDs or NDs, could overcome the inversion effect and distinguish the physically inverted (i.e., performed in the air) from artificially inverted movements when given sufficient dynamic information. Using the same experimental paradigm with different groups of participants, the current study not only replicated the previous finding where the extended visuomotor experience with inverted movements could remedy the inversion effect on perceiving human actions, but also extended the knowledge by showing that VDs engaged in longer fixations during the task to extract the dynamic information from the lower trunk for successful judgement. Interestingly, TDs demonstrated a similar fixation pattern as VDs, but they failed to use the same dynamic information to improve their sensitivity to the inversion. The comparisons between the three participant groups suggested two factors that could have contributed to VDs' ability to identify artificially inverted movements: the fixations at the lower trunk area to extract dynamic information from the kinematics, and the prior experience that facilitates the interpretation of the attended dynamic information.

With vertical dance, the dancers wear a climbing harness placed at the waist slightly above the iliac crest. They use this harness and a rope to be suspended in the air while performing various dance movements either in an upright or an inverted orientation (Figure 4). In a normal performance, the dancers would sway from the rope, resulting in the underlying dynamics of the overall movements being that of a pendulum:

$$\frac{d^2\theta}{dt^2} + \frac{g}{l}\sin\theta = 0$$

Where t is time, θ is the angle from the vertical to the pendulum, g is the gravitational constant, and l is the length of the line. Noticeably, only the gravitational force dictates the movement (kinematics) of the pendulum. In contrast, for dance movements performed on the ground, the dynamics of the trunk are governed by gravity and are subject to the influence of the force generated by the dancer’s limbs. The difference in the available force between the air and ground movements in the current study was manifested around the torso area. Since the vertical dancer is attached to the rope through a harness at the hip, any movement generated by arms and/or legs could result in a reactive motion of harness in the lower trunk area and, therefore, the kinematics of lower trunk motion specifies the pendulum dynamics of a particular dance movement. Conversely, when dancing on the ground, the lower trunk motion would be more constrained by leg movements without the influence of the arms and the harness and, therefore, the kinematics of lower trunk motion would specify the dynamics of gravity and that of the dancer’s self-initiated movement.

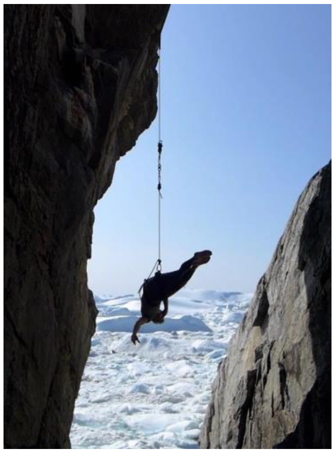

Figure 4. An example of a dancer performing a vertical dance movement while hanging upside down in the air.

Given the air and ground movements' unique dynamic signatures, the perceptual consequences of presenting these movements in an inverted orientation could also vary. For movements performed in the air, only the direction of the embedded gravitational force in the pendulum dynamics would be inverted. Because the professional VDs who recorded the original displays were able to maximally control their limb positions when performing movements in the air, movements of their limbs in the display did not contain sufficient information on the pendulum dynamics, requiring the observers to rely mainly on the movement of the lower torso area. In contrast, for movements performed on the ground, not only was the direction of gravity inverted, but, more importantly, the direction of the self-initiated force (e.g., using legs to push one off the ground) was also inverted. Therefore, the observers could rely on both movements of the torso and the limb to perform the judgment. Because of the richness of the dynamic information of the ground movements, participants of different experience levels could all readily identify the artificial inversion. Furthermore, compared to NDs, both VDs and TDs had significant dance experience, which includes education and knowledge of the role of the center of mass in postural control and dance performance (Ibuki et al., 2017; Munzert et al., 2019). Therefore, with a lack of experience or knowledge, NDs failed to pay attention to the lower torso area to extract relevant dynamic information from the air movements. Curiously, although both VDs and TDs were able to fixate on the lower torso area for the air movements, only VDs were able to successfully distinguish artificial inversion from physical inversion of the movements. What could have contributed to this discrepancy?

The mere knowledge of the information-rich area to attend does not guarantee effective information pick-up. The observations that 1) VDs and TDs had comparable eye movement patterns and 2) VDs had greater sensitivity to artificial inversion of air movements suggest that

VDs were able to utilize the kinematics of the lower trunk to extrapolate the embedded pendulum dynamics and leverage the dynamics to perform directional judgment, whereas TDs were not able to do so. In a recent meta-analysis, Silva and colleagues (2022) found that novices and experts did not have distinct visual search patterns in the context of sports but there were still distinct performance outcomes between the two groups. In other words, the general expertise (i.e., dance) may affect visual search strategies, and the area-specific expertise (i.e., vertical dance) dictates how effectively one could pick up and interpret the information obtained from the visual search process (Abernethy & Russell, 1987). While the current study did not address the question of how the dynamic information is interpreted, future studies could adopt a perturbation paradigm and systematically alter the kinematics of the lower torso area of the dancer in different directions to see how these changes would affect VDs' ability to perform the judgment. Similarly, because TDs are already familiar with the area around which they should attend to obtain the relevant dynamic information, future studies could also investigate the perceptual learning process through which TDs could learn how to interpret the dynamic information to perform the judgment.

In conclusion, the findings from this study highlight the complex interplay between perceptual expertise and extracting dynamic information from the kinematics (i.e., kinematic specification of dynamics) in the context of biological motion perception, particularly focusing on the inversion effect. Judgment results showed that extended visuomotor experience with vertical dance played a pivotal role in helping VDs overcome the inversion effect of PLDs. Shown through the eye tracking analysis, the ability of VDs to discern artificially inverted movements was underpinned by their adeptness in fixating on the lower trunk area to extract dynamic information critical for judgment. Intriguingly, while both VDs and TDs exhibited similar eye movement patterns, only VDs demonstrated heightened sensitivity to artificial inversion, suggesting that area-

specific expertise strongly influences information interpretation and subsequent perceptual outcomes. These insights underscore the nuanced nature of expertise-driven visual search strategies and emphasize the importance of considering domain-specific knowledge in understanding perceptual processes. Moving forward, future research could delve into the mechanisms underlying dynamic information interpretation and explore perceptual learning processes to enrich our understanding of expertise-mediated perceptual phenomena. By probing deeper into these issues, the intricacies of human perception and cognition can be unraveled and provide valuable guidelines in training and education.

Author Contributions

Xiaoye Michael Wang: Methodology, Software, Formal analysis, Investigation, Data curation, Writing – Original draft, Writing – Review & editing, Visualization, Project administration. **Zhichen Feng**: Investigation, Data curation. **Mingming Yang**: Investigation, Data curation. **Jing Samantha Pan**: Investigation, Data curation. **Margaret A. Wilson**: Methodology, Writing – Review & editing. **Qin Zhu**: Methodology, Investigation, Data curation, Writing – Review & editing, Project administration.

Competing Interests

The authors did not have any competing interests.

References

Abernethy, B., & Russell, D. G. (1987). The relationship between expertise and visual search strategy in a racquet sport. *Human Movement Science*, *6*(4), 283–319.

Barclay, C. D., Cutting, J. E., & Kozlowski, L. T. (1978). Temporal and spatial factors in gait perception that influence gender recognition. *Perception & Psychophysics*, *23*(2), 145–152. https://doi.org/10.3758/BF03208295

Cutting, J. E., & Kozlowski, L. T. (1977). Recognizing friends by their walk: Gait perception without familiarity cues. *Bulletin of the Psychonomic Society*, *9*(5), 353–356. https://doi.org/10.3758/BF03337021

Dalmaijer, E. S., Mathôt, S., & Van der Stigchel, S. (2014). PyGaze: An open-source, cross-platform toolbox for minimal-effort programming of eye-tracking experiments. *Behavior Research Methods*, *46*, 913–921.

Ganesan, A., Alakhras, M., Brennan, P. C., & Mello-Thoms, C. (2018). A review of factors influencing radiologists' visual search behaviour. *Journal of Medical Imaging and Radiation Oncology*, *62*(6), 747–757.

Ghose, U., Srinivasan, A. A., Boyce, W. P., Xu, H., & Chng, E. S. (2020). PyTrack: An end-to-end analysis toolkit for eye tracking. *Behavior Research Methods*, *52*, 2588–2603.

Ibuki, A., Mani, H., Takeda, K., Hasegawa, N., Yamamoto, K., Maejima, H., & Asaka, T. (2017). Characteristic relationship between the centre of pressure and the centre of mass during quiet standing in female ballet dancers. *Int Phys Med Rehab J*, *1*(2), 00009.

Johansson, G. (1973). Visual perception of biological motion and a model for its analysis. *Perception & Psychophysics*, *14*(2), 201–211. https://doi.org/10.3758/BF03212378

Johansson, G. (1976). Spatio-temporal differentiation and integration in visual motion perception: An experimental and theoretical analysis of calculus-like functions in visual data processing. *Psychological Research*, *38*(4), 379–393. https://doi.org/10.1007/BF00309043

Macmillan, N., & Creelman, C. (2005). *Detection Theory: A User's Guide*. Lawrence Erlbaum Associates.

Morey-Holton, E. R. (2003). The impact of gravity on life. In *Evolution on planet Earth* (pp. 143–159). Elsevier.

Munzert, J., Müller, J., Joch, M., & Reiser, M. (2019). Specificity of postural control: Comparing expert and intermediate dancers. *Journal of Motor Behavior*, *51*(3), 259–271.

Pavlova, M., & Sokolov, A. (2000). Orientation specificity in biological motion perception. *Perception & Psychophysics*, *62*(5), 889–899. https://doi.org/10.3758/BF03212075

Peirce, J., Gray, J. R., Simpson, S., MacAskill, M., Höchenberger, R., Sogo, H., Kastman, E., & Lindeløv, J. K. (2019). PsychoPy2: Experiments in behavior made easy. *Behavior Research Methods*, *51*(1), 195–203.

Runeson, S., & Frykholm, G. (1983). Kinematic specification of dynamics as an informational basis for person-and-action perception: Expectation, gender recognition, and deceptive intention. *Journal of Experimental Psychology: General*, *112*, 585–615. https://doi.org/10.1037/0096-3445.112.4.585

Shipley, T. F. (2003). The Effect of Object and Event Orientation on Perception of Biological Motion. *Psychological Science*, *14*(4), 377–380. https://doi.org/10.1111/1467-9280.24471

Silva, A. F., Afonso, J., Sampaio, A., Pimenta, N., Lima, R. F., Castro, H. de O., Ramirez-Campillo, R., Teoldo, I., Sarmento, H., González Fernández, F., & others. (2022). Differences in

visual search behavior between expert and novice team sports athletes: A systematic review with meta-analysis. *Frontiers in Psychology*, *13*, 1001066.

Stanislaw, H., & Todorov, N. (1999). Calculation of signal detection theory measures. *Behavior Research Methods, Instruments, & Computers*, *31*(1), 137–149.

Stevens, C., Winskel, H., Howell, C., Vidal, L.-M., Latimer, C., & Milne-Home, J. (2010). Perceiving dance schematic expectations guide experts' scanning of a contemporary dance film. *Journal of Dance Medicine & Science*, *14*(1), 19–25.

Sumi, S. (1984). Upside-down presentation of the Johansson moving light-spot pattern. *Perception*, *13*(3), 283–286.

Troje, N. F., Sadr, J., Geyer, H., & Nakayama, K. (2006). Adaptation aftereffects in the perception of gender from biological motion. *Journal of Vision*, *6*(8), 7–7.

Troje, N. F., Westhoff, C., & Lavrov, M. (2005). Person identification from biological motion: Effects of structural and kinematic cues. *Perception & Psychophysics*, *67*, 667–675.

Wang, X. M. (2023). *Movement-Based Human Identity Recognition: Analysis and Hypotheses*.

Wang, X. M., Wilson, M. A., Song, Y., Ma, G., Dong, M., & Zhu, Q. (2022). Extended visuomotor experience with inverted movements can overcome the inversion effect in biological motion perception. *Scientific Reports*, *12*(1), 17538.

Wolfe, J. M., & Horowitz, T. S. (2017). Five factors that guide attention in visual search. *Nature Human Behaviour*, *1*(3), 0058.

Zhu, Q., & Bingham, G. P. (2014). Seeing Where the Stone Is Thrown by Observing a Point-Light Thrower: Perceiving the Effect of Action Is Enabled by Information, Not Motor Experience. *Ecological Psychology*, *26*(4), 229–261. https://doi.org/10.1080/10407413.2014.957969
