# Supplementary material for: Experience modulates gaze behavior and the effectiveness of information pickup to overcome the inversion effect in biological motion perception
Source: PLoS One. 2025 Jan 28;20(1):e0317290. doi: 10.1371/journal.pone.0317290 (PMC11774361; doi:10.1371/journal.pone.0317290)
Supplement: Attachment — Submitted filename: Response to Reviewer Comments R1 V2.docx [file pone.0317290.s003.docx]

Reviewer #1: Review of "Experience Modulates Visual Search Strategies and the Effectiveness of Information Pickup to Overcome the Inversion Effect in Biological Motion Perception" by Wang et al.

Wang et al. performed a psychophysical experiment to examine the effect of training on judging biological motion. The authors used biological motion stimuli created from vertical dancers and analyzed participants' behavioral judgements and eye movements. The vertical dancers were sensitive to the figural artificial inversion and showed a specific pattern of fixations. The experiment was creative and well-designed. However, I have several concerns as described below.

Title (and throughout the paper)
The use of "visual search (strategies)" in this paper is misleading because the visual search literature is generally involved in situations where participants look for something. In this study participants were not explicitly asked to find an item among discrete items.
Response: We thank the reviewer for pointing out this terminology concern. We replaced the mention of “visual search (strategies)” with “gaze behaviors” or something along this line throughout the manuscript.

p. 6, 2nd para "Ponmanadiyil and Woolhouse (2018)"
The paper did not appear in the reference list.
Response: We added the reference.

p. 6, 3rd para "faster saccades"
This description is a bit misleading because the velocity of saccades was not analyzed in this study.

Response: We removed “faster saccades”.

p. 9 1st para
The phrase "The experiment was presented on ..." should probably read a phrase like "The experimental stimulus was presented on ..."

Response: We updated the text that now reads: “The experimental stimuli were presented on a Dell 22-inch monitor (E2222H)…”

p. 11, "Each group contained 10 trials with responses of yes for the displayed movement was artificially inverted and no for the movement was not inverted."
The phrases "was artificially inverted/was not inverted" are not clear to me.

Response: This part corresponds to an earlier description of the experimental methods that reads (p. 10): “The participants were told that the displayed movements could be artificially inverted from their canonical orientations, and their task was to identify whether the artificial inversion had occurred in an animation.” (We added “from their canonical orientations” in the revision to further clarify the experimental manipulation). Along the same line, we also edited the sentence in question here, which now reads: “Each group contained 10 trials with responses of yes for the displayed movement was artificially inverted from their canonical orientation and no for the movement was not inverted.”

pp. 11-12, "The locations of the fixation and the locations of each point in PLD were normalized based on the screen’s resolution, resulting in values between 0 and 1."
Readers will be also helpful to know how long the actual distance is. On p. 9, the authors say "... on a Dell 22-inch monitor (E2222H) with a 1680 × 1050 resolution." Then, does a normalized distance of 1 (maximal value) correspond to actual 22 inches?

Response: We thank the reviewer for this question.

The eye tracker measures eye movement based on the screen’s pixel coordinate system (we now added a clarifying sentence on p. 9 to clarify this point: “Raw eye tracking data, measured based on the screen’s pixel coordinate system (*x*-axis: 0 – 1680 pixels; *y*-axis: 0 – 1050 pixels), were collected using PyGaze…”). The points of each PLD were also presented in the screen’s pixel coordinate.

As we were drafting the response, we realized that the screen’s non-uniform scaling along the *x*- and *y*-axis could actually create a problem in interpreting the results (the normalized distance along the *x*-axis corresponds to a different number of pixels than the normalized distance along the *y*-axis). Therefore, in the revision, we performed the spatial analysis using the Euclidean distance measured in pixels.

We updated the methods that now reads:

“To examine this hypothesis, the pixel coordinates corresponding to the location of the fixation and the location of the pelvic center were recorded. The pelvic center was approximated as the midpoint between the two markers at the hip joints. To derive a spatial measure for the relationship between fixation and the PLD, the Euclidean distance (in pixels) between the pelvic center and the fixation point was calculated for each animation frame during the fixation period. Then, the mean distances, referred to as the mean Euclidean distance, across all fixation periods were calculated for every trial.”

We also updated the results section accordingly (p18-20). Because of the extent of the edit, we decided not to include the updated text here. Importantly, changing the dependent measure did not in any way change the findings.

p. 14, the 4th line from the bottom
"latter" should probably read "former."

Response: In this sentence:

“When the movement was performed in the air, VD (mean = 0.63, SE = 0.16) showed greater sensitivity than TD (mean = 0.04, SE = 0.16, *p* < 0.05) and ND (mean = 0.08, SE = 0.16, *p* = 0.06), albeit the latter only approached the traditional level of significance.”

The use of “latter” is appropriate because we are referring to the comparison between VD and ND, where the p value is not yet at the conventional level of significance.

p. 15, Figure 2
I am confused about that the baseline of bar graphs is d' = -1.0, not 0.0. d' = 0.0 is the reasonable baseline of sensitivity as the authors indicate on p. 11. Furthermore, on reading the vertical axis of the Congruent air condition, I found that mean VD = -0.7, TD & ND = ~-1.0. These values are unlikely to be consistent with the main text on p. 14, as the authors wrote "When the movement was performed in the air, VD (mean = 0.63, SE = 0.16) showed greater sensitivity than TD (mean = 0.04, SE = 0.16, p < 0.05) and ND (mean = 0.08, SE = 0.16, p = 0.06), ...." Am I missing something?

Response: We thank the reviewer for pointing out this oversight. We made a mistake when labeling the y-ticks, resulting in an incorrect mapping between the actual values of the bars and the y-tick labels. We now fixed this issue in Figure 2:

p. 18, "Spatial Analysis"
The authors performed an ANOVA with a factor of Joint on the normalized distances. I am concerned about the different joint conditions of normalized distances can be regarded as statistically independent from each other (i.e., as one of assumptions for performing an ANOVA) because the values for different joints were calculated based on the same gaze point at each time. For example, when normalized distances for the torso center becomes shorter, that for the head will be mathematically longer at that time since the same fixated gaze position was assumed. This kind of mathematical dependence might inflate the chance of rejecting the statistical null hypothesis. The authors may verify that performing ANOVAs on such a situation is valid or tolerable by citing appropriate statistical methodology articles or similar papers.
Response: We thank the reviewer for raising this valid concern regarding the statistical design. Comparing the spatial characteristics between fixation and various joints in the PLD indeed introduces dependencies among the dependent measures, which violates the assumption of independence required by ANOVA. To address this issue, we focused on the spatial relationship between the fixation point and the pelvic center, defined as the midpoint between the two hip joints depicted in the animations

We updated the methods section to delineate the rationale behind this decision (p. 12):

“The pelvic girdle including the hip joints plays an important role in the functional movement of dancers because it connects the upper and lower body and contributes to posture and balance control (Wanich-Romita & Romita, 2023). When performing dance movements midair while suspended in a harness, movement of the pelvis is restricted by the cable connecting the harness to the ceiling. In contrast, the pelvis is less constrained when performing the same dance movements on the ground. Therefore, the pelvic girdle may serve as an inherently information-rich area for the observer (especially dancers) to differentiate between air and ground movements. To examine this hypothesis, the pixel coordinates corresponding to the location of the fixation and the location of the pelvic center were recorded. The pelvic center was approximated as the midpoint between the two markers at the hip joints. To derive a spatial measure for the relationship between fixation and the PLD, the Euclidean distance (in pixels) between the pelvic center and the fixation point was calculated for each animation frame during the fixation period. Then, the mean distances, referred to as the mean Euclidean distance, across all fixation periods were calculated for every trial.”

By focusing on the pelvic center, we avoid examining fixation distances across multiple joints, thus maintaining the independence assumption required for ANOVA. As a result, our statistical model included only one between-subject factor (Experience) and one within-subject factor (Recording Type):

“Therefore, for the gaze event measures (i.e., saccade frequency, fixation frequency, and fixation duration) and the spatial measure (Euclidean fixation distance), a series of mixed-design ANOVA with one between-subject factor (Experience) and one within-subject factor (Recording Type) was used.”

We updated the results section accordingly (p. 19-20). Importantly, the revised statistical approach did not alter our original conclusion. Our findings demonstrate that, while typically developing individuals (TDs) know where to look, they do not necessarily know how to interpret the dynamic information. As a result, they exhibited poor sensitivity in detecting artificially inverted movements.

p. 16, line 8
"former" should probably read "latter."

Response: In this sentence:

“However, for the Complete condition, VD (mean = 1.32, SE = 0.15) had greater sensitivity than TD (mean = 0.85, SE = 0.14, *p* = 0.073) and ND (mean = 0.43, SE = 0.15, *p* < 0.001), albeit the former only approached the traditional level of significance.”

We did refer to the former (VD and TD) where the p-value is only approaching the conventional level of significance.

p. 20, first line
Typo: "Error! Reference source not found"

Response: We thank the reviewer for pointing out this issue. We were only able to see it after refreshing all the links in the document. We now updated the reference.

p. 21, Figure 3
"(x-axis)" is a bit misleading because it implies the coordinates of the screen for the PLD presentation.

Response: We removed “(x-axis)” from the caption.

Reviewer #2: This paper, which explores biomotion in the context of traditional versus vertical dance, is exceptionally well-written and clear. The findings advance our understanding of biological motion and the perceptual factors that inform it. I have provided only minor comments throughout, which should be straightforward to address. Congratulations on presenting a thoughtfully constructed and insightful study.

Response: We thank the reviewer for their encouragement and positive evaluation of our study. We incorporated the reviewer’s suggestions in the updated manuscript. Specifically we:

- Changed from “human” to “hominid” in the following sentence (p. 3): “As terrestrial animals, gravity has been an indispensable element in hominid’s evolutionary past, present, and future (Morey-Holton, 2003).”
- Edited the following sentence to avoid ambiguity (p.3): “Any human movement is subject to the influence of the entire force environment…”
- Used the entire terms when starting a sentence (p.3): “Point-light displays represent a person or an animated object using a series of moving points that are placed at different major joints” and (p.7): “Traditional dancers had an average of 12.25 years of dance experience…”
- Fixed a type (p.4): “For instance, Shipley (2003) used PLDs to present a person walking where the display was either in the movement’s canonical orientation or upside down.”
